# Single and Combined Supplementation of Resveratrol and Fumaric Acid on Energy Metabolism of *Litopenaeus vannamei*

**DOI:** 10.3390/ani16152338

**Published:** 2026-07-31

**Authors:** Jean Piraine Souza, Juan Rafael Buitrago Ramirez, Robson Matheus Marreiro Gomes, Alan Carvalho de Sousa Araújo, Wilson Wasielesky, Marcelo Borges Tesser, Sergiane Caldas Barbosa, José María Monserrat

**Affiliations:** 1 Laboratory of Functional Biochemistry of Aquatic Organisms (BIFOA), Institute of Oceanography (IO), Federal University of Rio Grande—FURG, Rio Grande 96210-030, RS, Brazil; jeanpiraine@gmail.com (J.P.S.); juanrafaelb25@gmail.com (J.R.B.R.); roobinho_matheus@hotmail.com (R.M.M.G.); alandesousa02@hotmail.com (A.C.d.S.A.); 2 Laboratory of Marine Shrimp Farming, Institute of Oceanography (IO), Federal University of Rio Grande—FURG, Rio Grande 96210-030, RS, Brazil; manow@mikrus.com.br; 3 Laboratory of Nutrition of Aquatic Organisms, Institute of Oceanography (IO), Federal University of Rio Grande—FURG, Rio Grande 96210-030, RS, Brazil; mbtesser@gmail.com; 4 Laboratory of Organic Compounds and Metals Analysis (LACOM), School of Chemistry and Food, Federal University of Rio Grande—FURG, Rio Grande 96203-900, RS, Brazil; sergianecaldas@furg.br; 5 Institute of Biological Sciences (ICB), Federal University of Rio Grande—FURG, Rio Grande 96203-900, RS, Brazil

**Keywords:** aquaculture, bioenergetics, organic acids, phytochemicals, zootechnical parameters

## Abstract

The study analysed the effects of dietary resveratrol supplementation, either alone or in combination, with fumaric acid on the zootechnical performance and bioenergetics parameters of juvenile *Litopenaeus vannamei*. A dose–response trial was conducted with 0, 10, 60, 110, and 160 mg·kg^−1^ of resveratrol. Subsequently, a second experiment was performed with shrimp from the control group and the best-performing resveratrol group. The treatments were control (C), fumaric acid 8.47 g·kg^−1^ (F), resveratrol (R) and R + F (RF). Growth performance (final weight gain (g), specific growth rate (SGR), feed conversion ratio (FCR), and protein efficiency ratio (PER)) and the concentration of energy-yielding molecules evaluated. Shrimp fed with 60 mg·kg^−1^ of resveratrol showed higher weight gain, SGR, protein and glucose concentrations in the muscle, increased PER, and lower FCR (ANOVA and Tukey HSD post hoc test (*p* < 0.05). Combined supplementation (RF) further enhanced growth performance and protein, glucose, and glycogen concentrations (*p* < 0.05) (ANCOVA test; *p* < 0.05). The zootechnical responses were also improved, where in 14 days the animals gained almost three grams of weight, with an FCR of 1.14 ± 0.03 and SGR of 6.99 ± 0.17% per day (*p* < 0.05). Overall, the combined supplementation of resveratrol and fumaric acid acted as an effective metabolic modulator, improving metabolic status of *L. vannamei*.

## Simple Summary

Feed represents the highest cost in shrimp farming. This study evaluated the effects of two dietary supplements, resveratrol (a natural polyphenol) and fumaric acid (an organic acid), to improve growth performance and metabolic efficiency in Pacific white shrimp (*Litopenaeus vannamei*). Two experiments were conducted over a total period of 42 d, shrimp were fed twice daily and the mean (±SD) values for water quality parameters were 6.03 ± 0.06, 28.64 ± 0.21, 29.20 ± 0.22, and 8.00 ± 0.12 for dissolved oxygen (mg/L), temperature (°C), salinity (ppt), and pH, respectively. First, we identified the optimal dose of resveratrol testing five inclusion levels (0, 10, 60, 110, and 160 mg·kg^−1^) during 28 d. Subsequently, we tested resveratrol (0 or the optimal dose of 60 mg·kg^−1^) alone or in combination with fumaric acid (8.47 g·kg^−1^) during 14 d. The results showed that the combined supplementation produced the best performance, significantly improving weight gain, feed conversion ratio, and the efficiency of protein and energy utilization (n = 3–15). These findings demonstrate the potential of combining resveratrol and fumaric acid as a metabolic modulator to enhance shrimp production in a more sustainable way.

## 1. Introduction

Feed represents the largest operating cost in shrimp farming, totaling between 40 and 65% of the final production costs associated with feed management. Therefore, the main challenge is to achieve a balance between a favourable environment, low-cost diet, and nutritional and energy quality that supports the optimal growth potential of the cultivated species [1,2].

*Litopenaeus vannamei* is the most widely cultured shrimp species worldwide, and nutritional strategies to improve feed efficiency remain a major research focus. Numerous studies have focused on understanding the main nutritional requirements for sustaining the maximum productive potential of this species, including nutritional requirements [3], effects of salinity and carbohydrate levels in metabolism [4] and the strategies employed to reach this objective [5,6].

From a bioenergetic perspective, carbohydrates, lipids, and proteins are macronutrients essential for the survival and development of a species. The nutritional requirements associated with each of these elements are influenced by multiple factors such as life stage, stocking density, water quality parameters, ingredient quality, biochemical profile of the macronutrient, digestibility, palatability, and total energy content of the feed [7,8].

For the shrimp *L. vannamei*, the inclusion level of carbohydrates in formulated diets is currently a maximum of 30% and this limitation is associated with the complexity of the carbohydrates used in the feed and its consequent digestibility, as well as the intrinsic metabolic characteristics of the animal, such as the low capacity for glycemic regulation in the face of high absorption rates in the intestinal tract and low sensitivity to insulin-like peptide (ILP), which signals glucose uptake pathways in tissues in response to increased intake [7,8,9,10].

Furthermore, the best lipid inclusion level in diets for these juvenile shrimps is between 10 and 12%, which is essential for the development of these animals, acting as hormone precursors, energy substrates, cellular structuring, and metabolic regulation [11,12,13,14,15]. Excess lipids in a formulation compromise pellet stability, increase the energy content of the feed, reduce feed intake by induce an early satiety, increase carcass lipid deposition, reduce protein efficiency, and decrease survival owing to increase oxidative damage and immune depression [1,15,16].

Proteins are the most expensive macronutrients in a formulated diet for white shrimp [17,18]. Dietary protein levels for this species typically range from 20 to 45% [1,19,20]. However, unlike other macromolecules, ingested protein cannot be stored for mobilization under conditions of need, limiting its use only to specific demands in the face of amino acid availability for tissues [10,20,21,22,23,24,25,26]. The protein-sparing effect contributes to the efficient utilization of the protein offered in the diet by improving the use of carbohydrates and lipids as energy substrates, whereas protein is allocated primarily to growth and other regulatory processes [10,27,28,29]. This concept has been evaluated through the manipulation of carbohydrate, lipid, and protein concentrations in diets by reducing protein concentration, aiming to determine the threshold for an effective protein-sparing effect [29,30,31,32].

The application of phytochemicals and biomolecules as potential metabolic modulators may facilitate the development of more economically and energetically efficient diets [28,33,34]. Several bioactive compounds, including resveratrol and fumaric acid, have been investigated as metabolic modulators in aquaculture [35,36,37,38,39,40,41]. The effects of these molecules on biological systems are highly dynamic and are associated with variables such as the dose–response pattern and target metabolic mechanisms in different organs, tissues, or cells through hormetic mechanisms [42,43].

Resveratrol is a polyphenol present mainly in the skin of dark grapes and their by-products, and its action in animals at the level of bioenergetic metabolism contributes to the reduction in the negative effects of hypercaloric diets by mimicking the condition of energy restriction through the activation of adenosine monophosphate-activated protein kinase (AMPK) and sirtuin 1 (SIRT-1), which represents one of the main axes of energy monitoring and signalling for catabolic processes of lipid and glycogen stores [40,44,45,46,47,48,49,50].

Resveratrol supplementation has improved growth performance and metabolic regulation in several fish species [40,51,52,53,54,55]. However, the administration of high doses of resveratrol to the diets of *Salmo salar* and *O. mikiss* reduced feed intake, thereby compromising growth performance and final productivity [46,56].

The inclusion of organic acids as nutritional supplements in formulated diets for aquatic organisms has been shown to improve the growth and productivity of *O. niloticus* and *L. vannamei* cultures [57,58,59,60,61]. Particularly, fumarate is an intermediate of the Krebs cycle and may enhance cellular energy metabolism [61]. Supplementation with fumaric acid increases intestinal villus height and width in *O. niloticus*, optimizing the absorption of essential nutrients from the diet [57].

Therefore, the objective of this study was to determine the optimal dietary dose of resveratrol, which expressed the best zootechnical response in juvenile *L. vannamei* shrimp and evaluate the effects of isolated and combined supplementation of resveratrol and fumaric acid on the protein, lipid, and carbohydrate levels of these animals to evaluate the potential for bioenergetic modulation of these supplements.

## 2. Materials and Methods

### 2.1. Shrimp Rearing

Experiments and analyses were conducted at the Laboratory of Functional Biochemistry of Aquatic Organisms (BIFOA) at the Marine Station of Aquaculture (EMA) of the Federal University of Rio Grande—FURG. Post-larvae (PL) of *L. vannamei* were obtained from Aquatech Ltda. (Natal, RN, Brazil) and maintained in a biofloc technology (BFT) nursery system until reaching a juvenile size of 0.5 ± 0.03 g.

### 2.2. Feed Preparation

The feed used in the experiments was the Guabitech line 40J (Sales, SP, Brazil) for juvenile marine shrimp, with 40% protein, 30% carbohydrates, 10% ether extract, 10% moisture, 4% crude fiber, 6% minerals, probiotics, antioxidants, and other supplements. Resveratrol (97% purity) used for feed preparation was obtained from Xtreme Manipulations Laboratory (Pelotas, RS, Brazil), and its purity was validated by high-performance liquid chromatography coupled with a diode array detector (HPLC-DAD), using resveratrol supplied by Sigma-Aldrich (PHR2201; St. Lous, MO, USA) as a standard [62] (Figure S1, Supplementary Material). Fumaric acid (INS 297) was acquired from Allimentari (São Paulo, SP, Brazil).

The feed samples were weighed and distributed in aluminium foil trays, ensuring that each contained 100 g of feed arranged in a single pellet layer to prevent overlap and eliminate empty spaces. Both resveratrol and fumaric acid were weighed and diluted in absolute ethanol (50 mL·kg^−1^ feed) and then sprayed onto the pellets according to their respective concentrations (mg·kg^−1^), whereas the controls were sprayed only with absolute ethanol [63,64]. A proportion of 50 mL of solution per kg of feed was used to avoid pellet saturation, residual accumulation of solution at the bottom of the tray, and consequent under-utilization of the supplements. Finally, the trays were placed in an oven at 40 °C for 24 h to allow the alcohol in the pellets to evaporate. Subsequently, the feeds were stored in a freezer at −20 °C.

### 2.3. Experimental Design

#### 2.3.1. Experiment 1: Resveratrol Dose–Response

**Figure 1:**
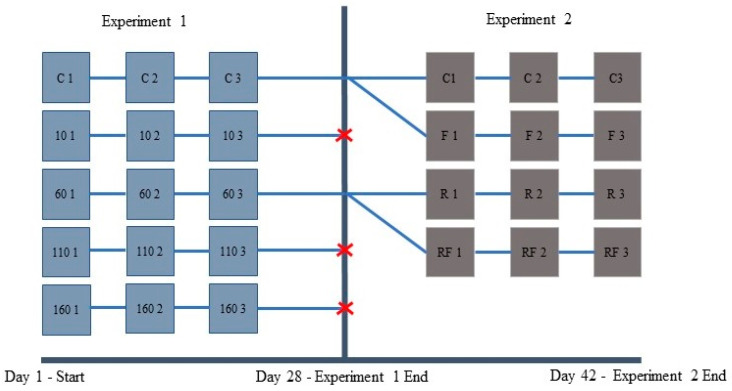
Schematic representation of experimental design. Experiment 1: dose–response with resveratrol (0, 10, 60, 110, 160 mg·kg^−1^) and 15 animals per tank. Experiment 2: with 5 animals per tank, a factorial combination of the best resveratrol dose (60 mg·kg^−1^) and fumaric acid (8.47 g·kg^−1^). C: control; F: fumaric acid; R: resveratrol; RF: resveratrol + fumaric acid.

For the dose–response experiment (experiment 1), 15 animals per experimental unit (0.5 ± 0.03 g) were stocked in 25 L of seawater (salinity 30 ± 1.42 ppt). Five groups were defined based on the concentration of resveratrol supplemented in 1 kg of feed: control group (no resveratrol supplementation), group 10 mg·kg^−1^, group 60 mg·kg^−1^, group 110 mg·kg^−1^, and group 160 mg·kg^−1^. Each treatment was evaluated in triplicate using three different tanks (Figure 1).

During the 28-day period, daily water quality parameters, such as total ammonia (mg.TAN·L^−1^), nitrite (mg·L^−1^), salinity (ppt), pH, temperature (°C), alkalinity (mg CaCO_3_·L^−1^), and dissolved oxygen (mg·L^−1^) were evaluated, with partial water changes to adjust the ideal conditions for the species and to remove uneaten feed and residues.

Weekly biometrics were performed to monitor zootechnical parameters, with feedings based on an expected feed conversion ratio of 1.25 and a weight gain of 1.5 g per week [65]. In subsequent weeks, the feed offered was based on the average weight (g), percentage of biomass, and tank temperature [66].

At the end of 28 d, all the animals were subjected to biometrics measurements and 10 animals from each tank of the control group and the best dose response group was restocked to Experiment 2 sequence. The remaining animals were euthanized for subsequent analyses (Figure 1).

#### 2.3.2. Experiment 2: Combined Supplementation

The results obtained by Silva et al. (2016) [60] served as a reference for defining the dose of 8.47 g·kg^−1^ of fumaric acid used in this experiment. Thus, the animals collected from each tank of the control group and the best performing-dose group in Experiment 1 were restocked in new experimental units with 5 animals per tank, filled with 25 L of seawater (salinity 30 ± 2 ppt).

With three tanks per group, four groups were defined based on the concentration of resveratrol and fumaric acid supplemented per kg of feed: control group (C), fumarate group (F), resveratrol group (R), and resveratrol + fumarate group (RF). The animals stocked in the tanks corresponding to C and F were from the control group of Experiment 1, whereas the animals in groups R and RF were from the best dose–response group in Experiment 1 (Figure 1). The groups R and C continued to receive the same treatment of Experiment 1, while the F and RF receive a fumaric acid supplementation.

Experiment 2 lasted 14 d, and the same water quality parameters as in Experiment 1 were evaluated daily. Partial water changes were performed to adjust the ideal conditions for the species and to remove uneaten feed and residues. Biometric measures were performed to monitor the zootechnical parameters and adjust the daily feeding rate [26]. Shrimp were euthanized at the end of the experimental period (Figure 1).

### 2.4. Analyses

The zootechnical parameters evaluated were total weight gain, feed conversion ratio (FCR), specific growth rate (SGR), and survival. For both experiment 1 and 2, animals were separated for analysis of protein efficiency ratio (PER), as well as sampling of hemolymph and tissues from the muscle and hepatopancreas for subsequent analyses of glucose, total cholesterol, total triglycerides, total proteins, and glycogen.

The zootechnical parameters were evaluated using the following formulas:

Formula 1. Survival:

$$S=\frac{Nf}{Ni\;}\;\times \;100$$

where

S: Survival (%·day^−1^).

Nf: Number of organisms at the end of the experiment.

Ni: Number of organisms at the beginning of the experiment.

Formula 2. Feed Conversion Ratio:

$$FCR=\frac{Do}{Bf}$$

where

FCR: Feed conversion ratio.

Do: Diet offered (g).

Bf: Final biomass (g).

Formula 3. Specific Growth Rate:

$$SGR=\left(\frac{lnWf-lnWi\;}{t}\right)\times 100$$

where

SGR: Specific growth rate (%·day^−1^).

lnWf: Natural logarithm of final weight (g).

lnWi: Natural logarithm of initial weight (g).

t: Time (days).

Formula 4: Total Weight Gain:

WGt=WGf−WGi

where

WGt: Total weight gain (g).

WGf: Final weight (g).

WGi: Initial weight (g).

Formula 5. Protein Efficiency Ratio:

$$PER=\frac{Gpt}{Pt}\times 100$$

where

PER: Protein efficiency ratio (%).

GPt: Total weight gain (g).

Pt: Total protein ingested (g).

#### 2.4.1. Sample Processing

Samples of muscle and hepatopancreas tissues and dry matter from the carcass were weighed in 2 mL microtubes and homogenized in homogenization buffer (Tris-base 20 mM, EDTA 1 mM, and KCl 150 mM), pH 7.4, with a 1:5 dilution of the sample in buffer. The homogenizer used was a sonicator at 50 kHz (QSonica Sonicators; Newton, CT, USA), with a 3 mm tip, in 30 s pulses for 3–5 min, and the samples were kept on ice throughout the process.

The dry matter in the carcass was evaluated at stocking and at the end of experiments 1 and 2. Animals were collected for weighing and verification of wet weight, dried in an oven at 60 °C for 4 h, and then weighed to obtain the dry weight, to calculate the dry matter.

#### 2.4.2. Analysis of Total Protein Concentration

To analyze the sample protein concentration in the muscle, hepatopancreas, hemolymph, and dry matter of the carcass, a Bioclin kit for total proteins (K031, Belo Horizonte, MG, Brazil) was used. In 1.5 mL microtubes, placed in duplicates, 7.5 μL of the homogenized sample, 7.5 μL of homogenization buffer in the blank microtubes, and 7.5 μL of albumin standard (40 mg·mL^−1^) in the standard tubes were added. To complete the reaction medium, 375 μL of Biuret monoreagent was added to all microtubes, which were then homogenized using a vortex for subsequent plate reading. In transparent 96-well plates with flat bottoms, 150 μL of the reaction medium was added to two wells per microtube of the sample, blank, or standard. Absorbance was measured at 550 nm using a Biotek microplate reader (Sinergy HT; Winooski, VT, USA), and protein concentration was calculated based on the absorbance obtained from the standard and expressed in mg·g^−1^ of wet tissue.

#### 2.4.3. Analysis of Glucose Concentration

To analyze the glucose concentration in the muscle, hepatopancreas, and hemolymph, the Labtest kit (Lagoa Santa, MG, Brazil) for glucose liquiform (133-1) was used. In 1.5 mL microtubes, placed in duplicate, 5 μL of the homogenized sample, 5 μL of homogenization buffer in the blank microtubes, and 5 μL of glucose standard (1 mg·mL^−1^) in the standard tubes were added. To complete the reaction medium, 500 μL of the monoreagent kit was added to all microtubes, which were then homogenized using a vortex and maintained in a water bath at 37 °C for 30 min for subsequent plate reading. In transparent 96-well plates with flat bottoms, 150 μL of the reaction medium was added, with two wells per microtube of sample, blank, or standard. The plate was incubated at 37 °C for 10 min before measuring the absorbance at 505 nm using a Biotek microplate reader (Sinergy HT). Glucose concentration was calculated based on the absorbance obtained from the standard and expressed as mg·g^−1^ of wet tissue.

#### 2.4.4. Analysis of Glycogen Concentration

For analysis of glycogen concentration in the muscle and hepatopancreas, an aliquot of 180 μL of homogenized sample and 20 μL of 5 M sodium hydroxide solution were added in 2 mL microtubes. The microtubes were then placed in a water bath at 100 °C for 30 min. Subsequently, 50 μL of supersaturated sodium sulfate (NaSO_4_) solution and 600 μL of absolute ethanol were added to the cooled samples, which were then centrifuged at 2000× *g* for 10 min. The resulting supernatant was discarded, and the pellets were placed in an oven at 60 °C for 60 min to volatilize the excess alcohol. The dried pellets were resuspended in 200 μL of distilled water. In 1.5 mL microtubes, placed in duplicates, 50 μL of the suspension obtained in the previous step, 50 μL of distilled water in the blank microtubes, and 50 μL of glucose standard (1 mg·mL^−1^) in the standard tubes were added. To complete the reaction medium, 120 μL of pure sulfuric acid (H_2_SO_4_) and 60 μL of 5% phenol were added to each microtube, which was then homogenized using a vortex for the subsequent plate reading. In transparent 96-well plates with flat bottoms, 150 μL of the reaction medium was added, with two wells per microtube of sample, blank, or standard. Absorbance was measured at 490 nm using a Biotek microplate reader (Sinergy HT) and glycogen concentration was calculated based on the absorbance obtained from the standard and expressed in mg·g^−1^ of wet tissue.

#### 2.4.5. Analysis of Triglyceride Concentration

For analyses of triglyceride concentration in the muscle, hepatopancreas, and hemolymph, the Bioclin kit for triglycerides (K117-2) was used. In 1.5 mL microtubes, placed in duplicate, 5 μL of the homogenized sample, 5 μL of homogenization buffer in blank microtubes, and 5 μL of triglyceride standard (1 mg·mL^−1^) in standard tubes were added. To complete the reaction medium, 500 μL of the kit monoreagent was added to each microtube, which was then homogenized using a vortex for the subsequent plate reading. In transparent 96-well plates with flat bottoms, 150 μL of the reaction medium was added, with two wells per microtube of sample, blank, or standard. Absorbance was measured at 500 nm using a Biotek microplate reader (Sinergy HT) and the triglyceride concentration was calculated based on the absorbance obtained from the standard and expressed in mg·g^−1^ of wet tissue.

#### 2.4.6. Analysis of Total Cholesterol Concentration

To analyze the total cholesterol concentration in the muscle, hepatopancreas, and hemolymph samples, a Labtest kit for Cholesterol Liquiform (76.1) was used. In 1.5 mL microtubes, placed in duplicate, 5 μL of the homogenized sample, 5 μL of homogenization buffer in blank microtubes, and 5 μL of cholesterol standard (1 mg·mL^−1^) in standard tubes were added. To complete the reaction medium, 500 μL of the kit monoreagent was added to all microtubes, which were then homogenized using a vortex for subsequent plate reading setup. In transparent 96-well plates with flat bottoms, 150 μL of the reaction medium was added, with two wells per microtube of sample, blank, or standard. Absorbance was measured at 510 nm using a Biotek microplate reader (Sinergy HT), and cholesterol concentration was calculated based on the absorbance obtained from the standard and expressed in mg·g^−1^ of wet tissue.

#### 2.4.7. Data Processing and Analysis

All statistical analyses were performed using Python (v3.10) with the statsmodels and SciPy libraries. For growth performance variables (weight gain, feed conversion ratio, specific growth rate, protein retention rate, and protein efficiency ratio), a varying-slope Analysis of Covariance (ANCOVA) was applied using initial body weight (IBW) as a covariate, allowing regression slopes to vary according to the presence or absence of resveratrol to control for baseline differences. Adjusted treatment means (Least-Squares Means) were evaluated at the overall covariate mean (1.6098 g) along with their respective 95% confidence intervals. Biochemical variables were analyzed using a two-way Analysis of Variance (ANOVA), with resveratrol and fumarate as the main fixed factors. Model assumptions were assessed based on residual analysis using the Shapiro–Wilk test for normality and Levene’s test for homogeneity of variance. Post hoc pairwise comparisons among treatment combinations were conducted using the Student–Newman–Keuls (SNK) test, using the residual mean square error (MSE) of the respective ANOVA/ANCOVA models. The weight gain (WG) and feed conversion ratio (FCR) were adjusted to a quadratic function by multiple linear regression, using dietary resveratrol level as the independent variable. The root of the derivative of the polynomial functions was calculated to estimate the optimal resveratrol values to maximize the weight gain and minimize the feed conversion ratio. The significance level was set at 0.05 (α = 0.05) for all statistical analysis.

## 3. Results

### 3.1. Water Quality Parameters

The parameters of water quality recorded over the 28 days of Experiment 1 are detailed in Table 1. No statistical differences (*p* > 0.05) were found in any of the parameters across the treatments.

The water quality parameters recorded during the 14-day period of Experiment 2 are presented in Table 2, where no significant differences (*p* > 0.05) were detected among treatments for any of the evaluated parameters.

### 3.2. Zootechnical and Bromatological Parameters

**Figure 2:**
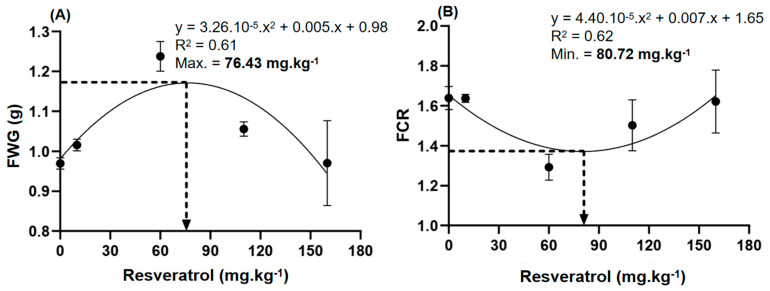
(**A**) Final weight gain in experiment 1, with data expressed as the mean ± standard error (n = 15). (**B**) Feed Conversion Ratio (FCR) in Experiment 1, with data expressed as the mean ± standard error (n = 15). Both models were statistically significant (*p* < 0.05).

The data on final weight gain (FWG) and FCR from Experiment 1 are presented in Figure 2, where the 60 mg·kg^−1^ treatment exhibited the highest weight gain among the experimental groups (Figure 2A) (*p* < 0.05), with the maximum weight gain estimated at 76.43 mg·kg^−1^ (R^2^= 0.6058) and the lowest FCR (Figure 2B), with the minimum FCR estimated at 80.72 mg·kg^−1^ (R^2^ = 0.5259), according to the estimation of the regression models (*p* < 0.05). The C, 10, 110, and 160 mg·kg^−1^ groups did not show significant differences (*p* > 0.05) in final weight gain and FCR (Figure 2A,B).

The specific growth rate (SGR) results from Experiment 1 are presented in Table 3, where the 60 mg·kg^−1^ group exhibited statistically higher (*p* < 0.05) values than the C, 10, and 160 mg·kg^−1^ groups, being statistically similar (*p* > 0.05) to the 110 mg·kg^−1^ group. For the protein efficiency ratio (PER), a significant increase (*p* < 0.05) was observed in the 60 mg·kg^−1^ group compared to the C, 10, and 160 mg·kg^−1^ groups, while there were no differences (*p* > 0.05) between the C, 10, 110, and 160 mg·kg^−1^ groups (Table 3). Additionally, there was no statistical differences (*p* > 0.05) in survival among the treatments at the end of Experiment 1 (Table 3).

In Experiment 2, the RF treatment exhibited the highest (*p* < 0.05) weight gain among all treatments (*p* < 0.05). The R group showed significantly higher (*p* < 0.05) weight gain than the C and F groups, whereas the C treatment showed the lowest (*p* < 0.05) weight gain (Table 4). The lowest feed conversion ratio (FCR) in Experiment 2 was also observed in the RF (*p* < 0.05) and the highest (*p* < 0.05) was in C group. No significant differences (*p* > 0.05) in FCR were detected among the F and R groups (Table 4).

The highest (*p* < 0.05) specific growth rate (SGR) in Experiment 2 was registered in the RF treatment (Table 4). The RF treatment exhibited the highest (*p* < 0.05) protein retention rate (PRR).

The protein efficiency ratio (PER) was significantly higher (*p* < 0.05) in the RF treatment than in the C and F treatments (*p* < 0.05), whereas among the C, F, and R groups, there were no significant differences (*p* > 0.05) (Table 4). No statistical differences (*p* > 0.05) in survival were observed among the treatments at the end of Experiment 2 (Table 4).

### 3.3. Total Protein Concentration

**Figure 3:**
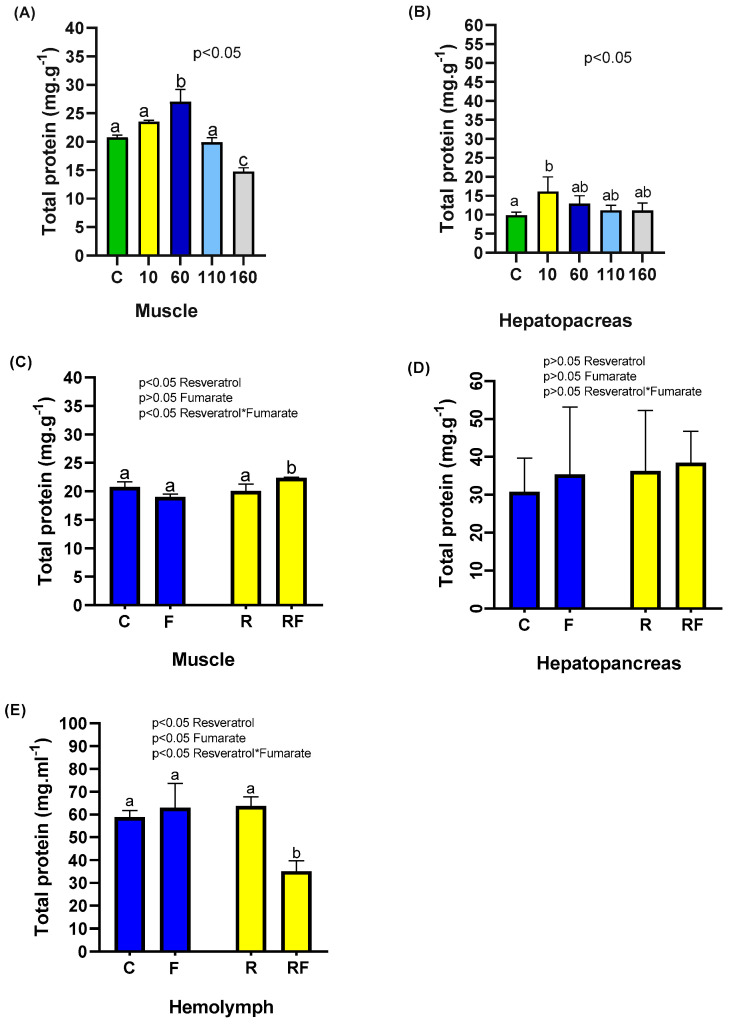
(**A**) Total protein content in the muscle (Experiment 1) (n = 3). (**B**) Total protein content in the hepatopancreas (Experiment 1) (n = 3). (**C**) Total protein content in the muscle (Experiment 2) (n = 3). (**D**) Total protein content in hepatopancreas (Experiment 2) (n = 3). (**E**) Total protein concentration in the hemolymph (Experiment 2) (n = 3). Treatments in Experiment 2: control group (C), fumaric acid group (F), resveratrol group (R), and resveratrol + fumaric acid group (RF). Different letters indicate statistically significant differences between treatments, as evaluated by the Tukey HSD (*p* < 0.05). In all cases, the data are expressed as mean ± standard error.

The analysis of total protein concentration in the muscle samples from Experiment 1 (Figure 3A), revealed significantly higher (*p* < 0.05) protein levels in the 60 mg·kg^−1^ group and significantly lower (*p* < 0.05) levels in the 160 mg·kg^−1^ treatment. In the hepatopancreas, an increase (*p* < 0.05) in protein concentration was observed in the 10 mg·kg^−1^ group compared to the control, but no significant differences (*p* > 0.05) were observed for the other treatments (Figure 3B).

In Experiment 2, muscle protein concentration was significantly higher in the RF treatment (*p* < 0.05), whereas no significant differences (*p* > 0.05) were observed among the R, F, and C treatment (Figure 3C). No significant differences (*p* > 0.05) in hepatopancreatic protein concentration were registered among all the treatments (Figure 3D).

In hemolymph samples from Experiment 2, the protein concentration was significantly lower (*p* < 0.05) in the RF treatment compared to the other treatments. No significant differences (*p* < 0.05) were observed among the R, F, and C treatments (Figure 3E).

### 3.4. Triglyceride Concentration

**Figure 4:**
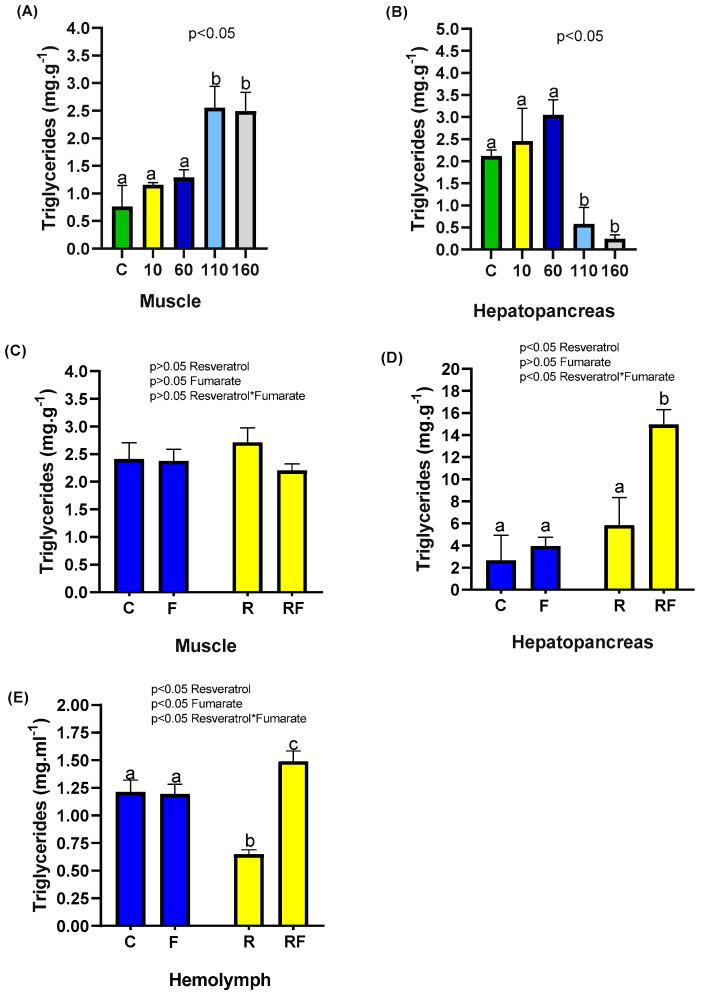
(**A**) Triglyceride concentration in muscle (Experiment 1) (n = 3) (**B**) Triglyceride concentration in hepatopancreas (Experiment 1) (n = 3). (**C**) Triglyceride concentration in muscle (Experiment 2) (n = 3). (**D**) Triglyceride concentration in hepatopancreas (Experiment 2) (n = 3). (**E**) Triglyceride concentration in hemolymph (Experiment 2) (n = 3). Treatments in Experiment 2: control group (C), fumaric acid group (F), resveratrol group (R), and resveratrol + fumaric acid group (RF). Different letters indicate statistically significant differences among treatments, as evaluated by the Tukey HSD test (*p* < 0.05). In all cases, the data are expressed as mean ± standard error.

The analysis of triglyceride concentration in muscle samples from Experiment 1 (Figure 4A) revealed significantly higher (*p* < 0.05) triglyceride levels in the 110 and 160 mg·kg^−1^ treatments, whereas no significant differences (*p* > 0.05) were observed between the C, 10, and 60 mg·kg^−1^ groups. The opposite pattern was observed for hepatopancreas samples from Experiment 1, with a significant reduction (*p* < 0.05) in triglyceride concentration in the 110 and 160 mg·kg^−1^ groups, while the C, 10, and 60 mg·kg^−1^ groups did not differ significantly (*p* > 0.05) (Figure 4B).

In Experiment 2, the treatments did not differ (*p* > 0.05) in muscle triglyceride concentrations (Figure 4C). Hepatopancreatic triglyceride concentration was significantly higher in the RF treatment than in the other treatments (*p* < 0.05), where triglyceride levels nearly twice those observed in the R treatment. There were no significant differences (*p* > 0.05) between the C, F, and R treatments (Figure 4D).

In the hemolymph samples from Experiment 2, the triglyceride concentration significantly decreased (*p <* 0.05) in the R group and increased (*p <* 0.05) in the RF group compared to the C and F groups, which did not differ significantly (*p >* 0.05) from each other (Figure 4E).

### 3.5. Total Cholesterol Concentration

**Figure 5:**
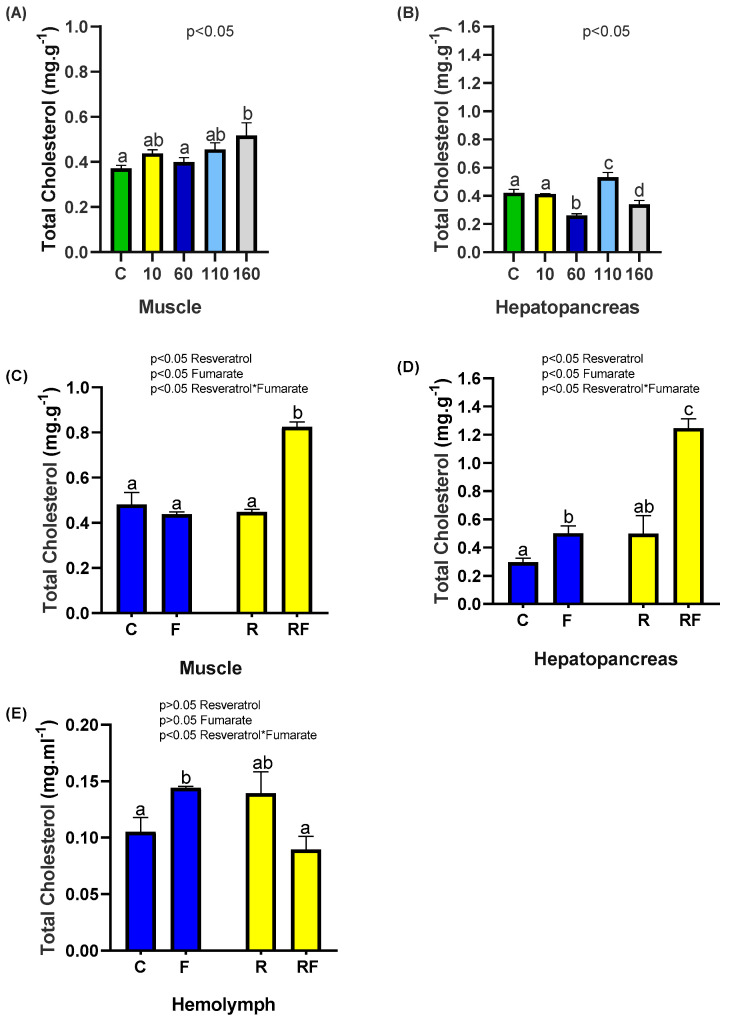
(**A**) Cholesterol concentration in the muscle (Experiment 1) (n = 3). (**B**) Cholesterol concentration in the hepatopancreas (Experiment 1) (n = 3). (**C**) Cholesterol concentration in the muscle (Experiment 2) (n = 3). (**D**) Cholesterol concentration in the hepatopancreas (Experiment 2) (n = 3). (**E**) Cholesterol concentration in the hemolymph (Experiment 2) (n = 3). Treatments in Experiment 2: control group (C), fumaric acid group (F), resveratrol group (R), and resveratrol + fumaric acid group (RF). Different letters indicate statistically significant differences among treatments, as evaluated by the Tukey HSD test (*p* < 0.05). In all cases, the data are expressed as mean ± standard error.

The analysis of cholesterol concentration in muscle samples from Experiment 1 (Figure 5A) revealed significantly higher (*p* < 0.05) cholesterol levels in the 160 mg·kg^−1^ group compared to the C and 60 mg·kg^−1^ groups. The 10 and 110 mg·kg^−1^ treatments did not differ significantly (*p* > 0.05) from the other groups. For hepatopancreas samples from experiment 1, cholesterol concentration was significantly lower (*p* < 0.05) in the 60 and 160 mg·kg^−1^ groups and higher in the 110 mg·kg^−1^ group (*p* < 0.05) compared to the C and 10 mg·kg^−1^ groups, which remained similar (*p* > 0.05) (Figure 5B).

In Experiment 2, the highest muscle cholesterol concentration was observed in the RF treatment, with values approximately twice those observed in the R treatment (*p* < 0.05), while the C, F, and R treatments were not significantly different (*p* > 0.05) (Figure 5C). In the hepatopancreas, the cholesterol concentration in the RF group was higher (*p* < 0.05) than that in the other treatment groups. The F treatment also exhibited significantly higher cholesterol levels than the C treatment (*p* < 0.05), and no significant difference (*p* > 0.05) was observed between the F and R treatments (Figure 5D).

In the hemolymph samples from Experiment 2, cholesterol concentration was significantly higher (*p* < 0.05) in the F treatment when compared with C and RF treatments. No significant differences (*p* > 0.05) were observed among the F and R treatments and among C and RF treatments (Figure 5E).

### 3.6. Glucose Concentration

**Figure 6:**
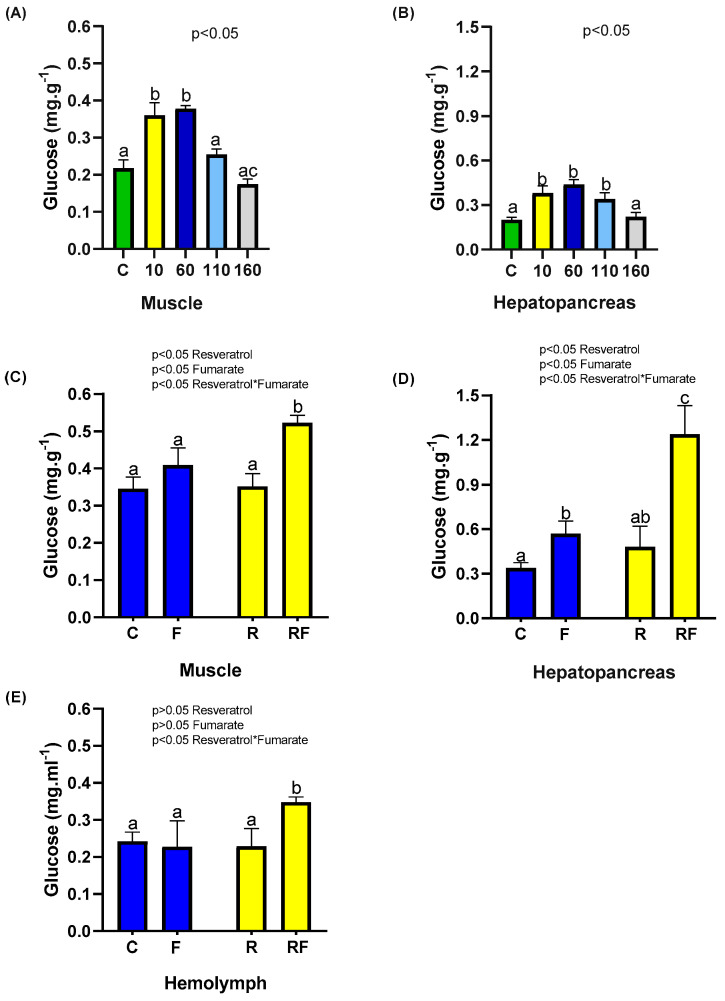
(**A**) Glucose concentration in the muscle (Experiment 1) (n = 3). (**B**) Glucose concentration in the hepatopancreas (Experiment 1) (n = 3). (**C**) Glucose concentration in the muscle (Experiment 2) (n = 3). (**D**) Glucose concentration in hepatopancreas (Experiment 2) (n = 3). (**E**) Glucose concentration in the hemolymph (Experiment 2) (n = 3). Treatments in Experiment 2: control group (C), fumaric acid group (F), resveratrol group (R), and resveratrol + fumaric acid group (RF). Different letters indicate statistically significant differences among treatments, as evaluated by the Tukey HSD test (*p* < 0.05). In all cases, the data are expressed as mean ± standard error.

The analysis of glucose concentration in muscle samples from Experiment 1 (Figure 6A) revealed significantly higher (*p* < 0.05) glucose levels in the 10 and 60 mg·kg^−1^ treatments than in the other treatments. The treatments C, 110, and 60 mg·kg^−1^ showed no statistical differences (*p* > 0.05). For hepatopancreas samples from Experiment 1, glucose concentration was significantly higher (*p* < 0.05) in the 10, 60, and 110 mg·kg^−1^ treatments compared to the C and 160 mg·kg^−1^ groups, which did not differ significantly (*p* > 0.05) from each other (Figure 6B).

In Experiment 2, the highest glucose concentration in the muscle was observed in the RF treatment (*p* < 0.05), whereas no statistical differences (*p* > 0.05) were observed among the C, F, and R groups (Figure 6C). Hepatopancreatic glucose concentration was highest (*p* < 0.05) in the RF treatment, with values approximately twice those observed in the C, F, and R treatments, which did not show significant differences (*p* > 0.05) (Figure 6D).

In the hemolymph samples from Experiment 2, glucose concentration was significantly higher (*p* < 0.05) in the RF treatment than in the other treatments. No significant differences (*p* > 0.05) were observed among the C, F, and R treatments (Figure 6E).

### 3.7. Glycogen Concentration

**Figure 7:**
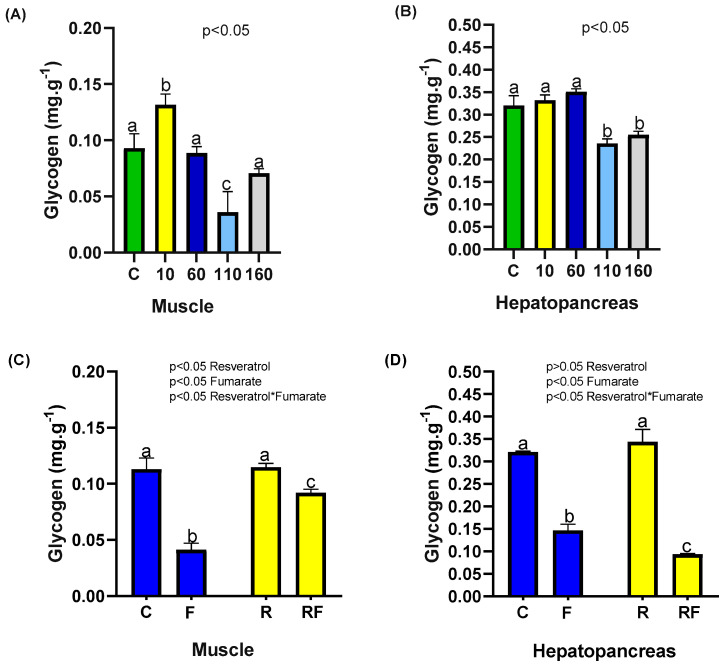
(**A**) Glycogen concentration in the muscle (Experiment 1) (n = 3). (**B**) Glycogen concentration in hepatopancreas (Experiment 1) (n = 3). (**C**) Glycogen concentration in muscle (Experiment 2) (n = 3). (**D**) Glycogen concentration in hepatopancreas (Experiment 2) (n = 3). Treatments in Experiment 2: control group (C), fumaric acid group (F), resveratrol group (R), and resveratrol + fumaric acid group (RF). Different letters indicate statistically significant differences among treatments, as evaluated by the Tukey HSD test (*p* < 0.05). In all cases, the data are expressed as mean ± standard error.

The analysis of glycogen concentration in muscle samples from Experiment 1, (Figure 7A) revealed significantly higher (*p* < 0.05) glycogen levels in the 10 mg·kg^−1^ treatment (*p* < 0.05) and significantly lower (*p* < 0.05) levels in the 110 mg·kg^−1^ treatment compared with the other treatments (C, 60, and 160 mg·kg^−1^), which did not differ significantly (*p* > 0.05) from one another. For hepatopancreas samples from Experiment 1, a significant reduction (*p* < 0.05) in glycogen concentration was observed in the 110 and 160 mg·kg^−1^ treatments, whereas the C, 10, and 60 mg·kg^−1^ groups did not differ significantly (*p* > 0.05) from each other (Figure 7B).

In Experiment 2, muscle glycogen concentration was significantly lower (*p* < 0.05) in the F and RF treatments, with F being significantly lower (*p* < 0.05) than RF, whereas C and R were not significantly different (*p* > 0.05) (Figure 7C). In the hepatopancreas, a pattern similar to that observed in muscle tissue, the glycogen concentration in the F and RF treatments was statistically lower (*p* < 0.05) than that in the other treatments, and the RF treatment exhibited the lowest value (*p* < 0.05). No significant differences (*p* > 0.05) were observed between the C and R treatments (Figure 7D).

## 4. Discussion

From a practical perspective, including resveratrol and fumaric acid in experimental feeds, this method proved to be efficient. Several studies have explored the spray-coated additives method in aquaculture feed, which reinforced its efficiency and practicality [64,67,68]. In contrast, the inclusion of bioactive compounds and nutraceuticals directly in feed formulations that need processing and extrusion can substantially alter the properties of these biomolecules, most of which are thermosensitive [69,70].

Many studies have indicated that resveratrol is an effective modulator of bioenergetic metabolism in rodents, birds, swine, and fish; however, relatively few studies have investigated crustaceans and other invertebrates [36,45,54,71,72]. Liu et al. (2021) observed that inclusion levels of 80 and 120 mg·kg^−1^ of resveratrol in diets formulated for the shrimp *L. vannamei* positively influence the antioxidant defense system of these animals under stress conditions induced by high levels of ammonia in the environment [51]. For the fish *Channa argus*, inclusion between 75 and 125 mg·kg^−1^ of resveratrol contributed to the best results in SGR and final weight gain [73]. Therefore, based on the data obtained in the dose–response experiment, where the best final weight gain, SGR, and FCR were observed in the 60 mg·kg^−1^ treatment compared with the other groups (Figure 1 and Table 3) with maximum growth at 76.53 mg·kg^−1^ and FCR at 80.72 mg·kg^−1^. This differs from a previous study that suggested that supplementation with 400 mg·kg^−1^ of resveratrol in extruded diets for white shrimp juveniles (0.4 ± 0.02 g) significantly increase the final weight gain (g) and SGR [6]. The thermal stability of resveratrol can be compromised at high temperatures over prolonged periods [74], suggesting that the addition of 400 mg·kg^−1^ in an extruded diet may not represent a final dose of 400 mg·kg^−1^.

Salomão et al. (2019) consider that resveratrol promotes growth by modulating amino acid metabolism in juvenile pacu (*Piaractus mesopotamicus*) [75], while Wilson et al. (2015) reported a positive effect of resveratrol supplementation on weight gain and reduced muscle protein degradation in flounder (*Paralichthys lethostigma*) [76]. As observed in Experiment 1, 60 mg·kg^−1^ of resveratrol may have contributed to increasing muscle protein and PER in *L. vannamei* shrimp, indicating that this dose contributed to biomass construction.

Menoyo et al. (2019) and Zhang et al. (2017) identified a negative effect of supplementation with high doses of resveratrol (above 400 mg·kg^−1^) in diets for *S. salar* and *M. amblycephala* on feed intake and consequent growth inhibition, possibly due to reduced feed palatability [46,54]. The inclusion of 300 mg·kg^−1^ of resveratrol in the diet of *O. mykiss* contributes to reduced feed intake due to premature satiety [52,53]. In contrast, fumaric acid has been widely used as a supplement in pig and poultry production, where it enhances dietary palatability [77,78]. In this context, fumaric acid may reduce the possible negative effects associated with resveratrol, in addition to increasing diet palatability and improving nutrient digestibility.

Regarding fumaric acid, diets supplemented with 8.47 g·kg^−1^ significantly improve digestion, weight gain, SGR, and FCR in *L. vannamei* juveniles compared to other organic acids such as succinate, acetate, butyrate, propionate, lactate, and citrate [60]. Additionally, supplementation of tilapia (*O. niloticus*) diets with 15 g·kg^−1^ fumaric acid promoted a significant increase in final weight gain, mainly by positively influencing the digestibility and absorptivity of macronutrients in the intestinal epithelium, as well as contributing as an intermediate in the Krebs cycle in glycolytic metabolic reactions [57]. For the shrimp *P. monodon*, a positive effect was observed with a supplementation of 10 g·kg^−1^ of butyrate, succinate, and fumarate on survival, feed intake, productivity, and nutrient retention efficiency [59]. The results of Experiment 2 do not show an effective effect of the fumaric acid 8.47 g·kg^−1^ dose supplement alone, where the C and F groups demonstrated equal values for weight gain, SGR, and FCR along 14 days of the experimental period. The metabolic effect of this supplementation can be estimated by the short experimental period if compared with 45 trial days, as reported by Silva et al. (2016) [60].

In Experiment 2, the combined supplementation of fumaric acid and resveratrol (RF treatment) showed a positive effect on weight gain, SGR and muscle protein coupled with reduced protein concentration in the hemolymph, indicating the flow of absorbed nutrients from circulation to tissues. The higher PER in RF treatment also reinforced the muscle concentration results, demonstrating that the ingested protein is retained in tissues and spared from energy demand, as previously reported in fish supplemented with resveratrol. [75,76].

The inclusion of resveratrol in experimental diets for rats enhances and optimizes the uptake of fatty acids from circulation, mitochondrial biogenesis, and β-oxidation in the skeletal muscle [79]. In tilapia (*O. niloticus*) and zebrafish (*Danio rerio*), resveratrol modulates lipid metabolism, reduces circulating and hepatic triglyceride concentrations, and attenuates the damage associated with high-calorie diets [55,80]. Additionally, in the liver of carp (*Cyprinus carpio*) it was observed that resveratrol effectively modulated triglyceride metabolism by inhibiting metabolic pathways associated with lipogenesis [81].

Crustaceans are generally incapable of de novo cholesterol synthesis, which restricts the uptake of this macronutrient exclusively from the diet. Cholesterol acts as a precursor for steroid hormones, which directly modulate the molting and reproduction cycles. Additionally, cholesterol is essential for maintaining the structural integrity of the plasma membrane, along with phospholipids, which mitigates the adverse effects of thermal and osmotic stress [82,83,84]. Numerous studies have shown that resveratrol modulates lipid metabolism by reducing the circulating and tissue concentrations of triglycerides and cholesterol [36,52,85]. Thus, as observed in the results of Experiment 1, the dose with the best zootechnical response (60 mg·kg^−1^) effectively contributed to the reduction in hepatopancreatic cholesterol concentration, as did the highest dose (160 mg·kg^−1^). This indicates that cholesterol may be mobilized from the hepatopancreas to meet tissue anabolic demands. However, this was not linearly reflected in final weight gain, as the reduction in hepatopancreatic cholesterol concentration and increase in muscle cholesterol in the 160 mg·kg^−1^ group did not effectively contribute to final weight gain in this group, whereas in the 60 mg·kg^−1^ group, with higher final weight gain, no significant increase in muscle cholesterol was observed. These results suggest that the RF treatment induced a metabolic trade-off in the energy-yielding substrates. The higher muscle protein content of Experiment 2 (Figure 3C) and PER was accompanied by a reduction in glycogen reserves in the same tissue (Figure 7C). This pattern suggests that carbohydrates were preferentially utilized to meet increased energy demands associated with enhanced growth, thereby promoting a protein-sparing effect. Furthermore, the increased triglyceride and cholesterol concentrations observed in the hepatopancreas of the RF treatment (Figure 4D and Figure 5D), together with the higher muscle cholesterol levels (Figure 5C), suggest that these lipids were preferentially allocated to the synthesis of cellular membranes, lipoproteins, and steroid hormone precursors.

In Experiment 2, the significant increase in cholesterol concentration in the muscle and hepatopancreas in the RF group, associated with reduced circulating concentration, highlights the efficiency of combined supplementation for the uptake of circulating cholesterol, which likely influences muscle gain. However, as an essential component of lipoproteins that promotes triglyceride absorption from circulation, increased cholesterol concentration in circulation is normally associated with increased tissue triglyceride concentrations [86].

The ability of resveratrol to modulate glycolytic metabolism in species of interest in aquaculture is widely reported However, no concrete investigations have been conducted regarding the application of this tool in the nutrition enhancer of *L. vannamei* shrimp. Liu et al. (2022), in *Micropterus salmoides*, and Shi et al. (2018), in *Megalobrama amblycephala*, demonstrated that diets supplemented with resveratrol significantly boost the activation of the AMPK/SIRT-1 metabolic pathway, which increases insulin sensitivity and mobilization of hepatic glycogen reserves as an energy substrate [40,87]. Also, resveratrol improves circulating glucose uptake by increasing the mobilization of glucose channels in both the muscle (GLUT 2) and liver (GLUT 4), aiding in maintaining glycemic homeostasis [43].

Accordingly, the concentration of glucose in muscle and hepatopancreas of animals in Experiment 1 showed a positive correlation between higher final weight gain and glucose concentration in these tissues. When analyzing the response in glycogen concentration in both tissues at intermediate doses, low accumulation was observed even with the highest weight gain in the 60 mg·kg^−1^ treatment, suggesting active glycolytic metabolism in utilizing glucose absorbed from circulation for energy supply.

In Experiment 2, supplementation with fumaric acid and the combination of supplements directly influenced glycolytic metabolism in cultured animals. In the RF group, the increase in glucose concentration in the hemolymph, muscle, and hepatopancreas, associated with reduced muscle and hepatopancreatic glycogen, indicated efficient glycolytic metabolism, especially when associating this metabolic pattern with the best zootechnical results, muscle protein gain, and PER obtained in this treatment. Additionally, it demonstrates the ability of resveratrol and fumaric acid to modulate the concentration of energy metabolites of *L. vannamei* shrimp.

Overall, the metabolic influence of RF treatment seems to be related to a trade-off between different macromolecules: glycogen channeled through the glycolytic pathway to produce ATP explains the lower levels registered in the R and RF treatments (Figure 7C,D), promoting a protein-sparing effect in muscle that favour protein deposition in this tissue (Figure 3C). By the other way, triglyceride levels remained unaffected in RF treatment (Figure 4C). Taken together, these results point to the fact that RF treatment influenced catabolism, being glycolysis the predominant pathway, thereby favoring the protein-sparing effect and improved growth.

## 5. Conclusions

Dietary supplementation with 60 mg·kg^−1^ resveratrol was identified as the optimal dose for improving growth performance in juvenile *L. vannamei*. Additionally, resveratrol exhibited a hormetic dose–response pattern, reinforcing the beneficial effects at intermediate concentrations. Furthermore, the combined supplementation of resveratrol and fumaric acid effectively modulated the bioenergetic metabolism of *L. vannamei* shrimp. This effect was particularly evident in the RF treatment in the final weight gain, SGR, and FCR. Over the 14-day experimental period, shrimps with RF treatment gained nearly three grams of weight, with an FCR of 1.14 and SGR of 7% per day. Although these findings demonstrate the positive effects elicited by RF treatment, the metabolic pathways influenced by resveratrol and the combination of resveratrol and fumaric acid require further investigation. In this context, it is important to mention that transcription factors, such as nuclear factor erythroid 2-related factor 2 (Nrf2), which has been described in crustaceans [88] to regulate the expression of antioxidant genes, also induce genes involved in ATP-generating pathways including glycolysis, the Krebs cycle and oxidative phosphorylation [89].

## Figures and Tables

**Table 1:** Water quality parameters for Experiment 1. Data are presented as mean ± standard error (n = 3). No significant differences were observed among treatments for any of the evaluated parameters (*p* > 0.05). C: control group.

Parameters	Treatments (mg of Resveratrol·kg^−1^ of Feed)				
	C	10	60	110	160
Dissolved oxygen (mg·L^−1^)	5.99 ± 0.08	6.03± 0.06	6.02 ± 0,03	5.97 ± 0.09	6.12 ± 0.08
Temperature (°C)	28.60 ± 0.03	28.30 ± 0.02	28.80 ± 0.02	28.80 ± 0.03	28.70 ± 0.05
Salinity (ppt)	29.52 ± 0.10	28.90 ± 0.12	29.1 ± 0.09	29.30 ± 0.18	29.20 ± 0.15
pH	7.91 ± 0.05	8.01 ± 0.06	7.89 ± 0.08	7.99 ± 0.05	8.20 ± 0.05
Ammonia (mg TAN·L^−1^)	0.32 ± 0.19	0.28 ± 0.21	0.29 ± 0.25	0.51 ± 0.26	0.37 ± 0.24
Nitrite (mg·L^−1^)	0.59 ± 0.16	0.62 ± 0.15	0.51 ± 0.25	0.49 ± 0.19	0.41 ± 0.17
Alkalinity (mg CaCO_3_·L^−1^)	143.25 ± 8.90	155.10 ± 11.60	140.52 ± 5.62	149.69 ± 6.52	156.00 ± 7.66

**Table 2:** Water quality parameters for Experiment 2. Treatment groups are designed as follows: control (C), fumaric acid (F), resveratrol (R), and fumaric acid + resveratrol (RF). Data are presented as mean ± standard error (n = 3). No significant differences were observed among treatments for any of the evaluated parameters (*p* > 0.05).

Parameters	Treatments			
	C	F	R	RF
Dissolved Oxygen (mg·L^−1^)	6.01 ± 0.04	6.08 ± 0.02	5.89 ± 0.10	5.92 ± 0.07
Temperature (°C)	28.2 ± 0.01	27.90 ± 0.06	28.10 ± 0.04	28.00 ± 0.03
Salinity (ppt)	29.00 ± 0.05	28.90 ± 0.09	28.60 ± 0.12	29.20 ± 0.09
pH	8.23 ± 0.14	7.98 ± 0.16	8.02 ± 0.12	8.03 ± 0.10
Ammonia (mg TAN·L^−1^)	0.12 ± 0.06	0.15 ± 0.05	0.18 ± 0.05	0.16 ± 0.04
Nitrite (mg·L^−1^)	0.29 ± 0.23	0.28 ± 0.18	0.32 ± 0.16	0.19 ± 0.12
Alkalinity (mg CaCO_3_·L^−1^)	152.25 ± 15.32	150.10 ± 12.61	149.20 ± 9.62	149.12 ± 8.52

**Table 3:** Zootechnical parameters measured in Experiment 1: Specific growth rate (SGR) (n = 3), Protein efficiency ratio (PER) (n = 3), and Survival (%) (n = 15). Different letters within the same row indicate statistically significant differences (*p* < 0.05) among treatments, as evaluated by the Tukey HSD test. For all variables, the data are expressed as the mean ± standard error. C: control group.

Parameters	Treatments (mg of Resveratrol·kg^−1^ of Feed)				
	C	10	60	110	160
SGR (%·day^−1^)	3.59 ± 0.03 ^a^	3.89 ± 0.03 ^a^	4.49 ± 0.05 ^b^	4.07 ± 0.09 ^ab^	3.86 ± 0.22 ^a^
PER	1.53 ± 0.03 ^a^	1.53 ± 0.01 ^a^	1.94 ± 0.05 ^b^	1.63 ± 0.05 ^ab^	1.55 ± 0.09 ^a^
Survival (%)	100 ± 0 ^a^	100 ± 0 ^a^	100 ± 0 ^a^	100 ± 0 ^a^	100 ± 0 ^a^

**Table 4:** Zootechnical parameters measured in Experiment 2: Weight gain (n = 15), Feed conversion ratio (FCR) (n = 15), Specific growth rate (SGR) (n = 15), and Protein efficiency ratio (PER) (n = 3). Treatment groups are designed as follows: control (C), fumaric acid (F), resveratrol (R), and fumaric acid + resveratrol (RF). Statistical analysis was performed using analysis of covariance (ANCOVA) with least-square means (LS-Means) ± standard error for growth performance variables. Different letters within the same row indicate statistically significant differences among treatments, evaluated by the Student-Newman-Keuls (SNK) test (*p* < 0.05).

Parameters	Treatments			
	C	F	R	RF
Weight gain (g)	2.15 ± 0.33 ^d^	2.43 ± 0.33 ^c^	2.61 ± 0.14 ^b^	3.245 ± 0.14 ^a^
FCR	1.42 ± 0.26 ^a^	1.24 ± 0.26 ^b^	1.13 ± 0.11 ^b^	0.94 ± 0.11 ^c^
SGR (%·dia^−1^)	6.07± 0.68 ^c^	6.67 ± 0.68 ^b^	6.84 ± 0.29 ^b^	7.90 ± 0.29 ^a^
PER	1.84 ± 0.31 ^d^	2.05 ± 0.31 ^c^	2.22 ± 0.13 ^b^	2.54 ± 0.13 ^a^
Survival (%)	100 ± 0 ^a^	100 ± 0 ^a^	100 ± 0 ^a^	100 ± 0 ^a^

## Data Availability

The raw data supporting the conclusions of this article will be made available by the authors on request.

## Supplementary Materials

The following supporting information can be downloaded at: https://www.mdpi.com/article/10.3390/ani16152338/s1.
