# Supplementary material for: Single and Combined Supplementation of Resveratrol and Fumaric Acid on Energy Metabolism of Litopenaeus vannamei
Source: Animals (Basel). 2026 Jul 31;16(15):2338. doi: 10.3390/ani16152338 (PMC13463505; doi:10.3390/ani16152338)
Supplement: Supplementary file 1 [file animals-16-02338-s001.zip › animals-4400565-supplementary.pdf]

Supplementary Material

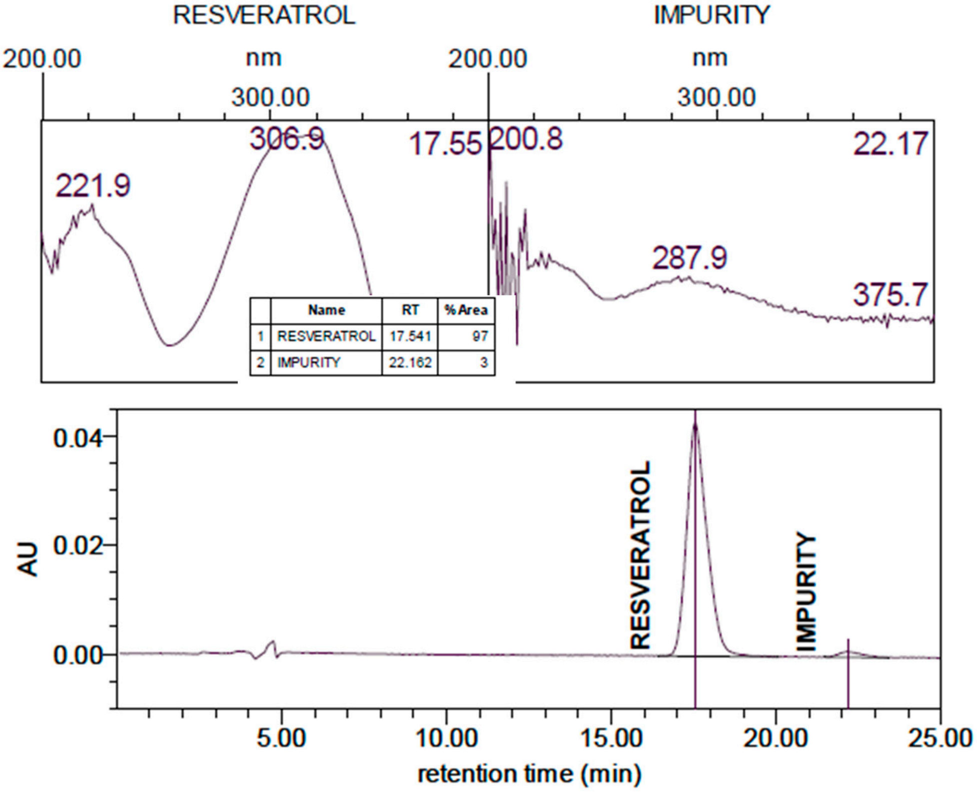

Figure S1. HPLC-DAD chromatogram of resveratrol standard showing the peaks detected and their respective UV–Vis spectrum (inset: table with the percentage of the peak area) (Analysis conditions: Analyses were performed using an LC apparatus consisting of a column, Synergy Fusion C18 4 µm (25 cm × 4.6mm i.d.), a Waters 600 model pump associated with a Waters 2996 Photodiode Array Detector, and a Rheodyne loop injector. The mobile phase was acetonitrile:water containing 0.2% formic acid (25:75, v/v). The UV detector wavelength was set from 200 to 400 nm. The flow-rate was 0.8 mL/min and the injection volume was 20 µL.
